# Appendix to "A Dissertation on the Effects of Malaria, and Other Malign Atmospherical Influences, in the Production of Febrile Diseases of Various Types and Degrees of Intensity."

**Published:** 1833-01

**Authors:** Andrew Blake

**Affiliations:** Physician to the General Lunatic Asylum near Nottingham; late of his Majesty's 7th Regiment of Dragoon Guards, and author of "an Essay on Delirium Tremens," &c.


### EFFECTS OF MALARIA, &c.

*Appendix to "A Dissertation on the Effects of Malaria, and other Malign Atmospherical Influences, in the Production of Febrile Diseases of various Types and Degrees of Intensity."* By Andrew Blake, M.D., M.R.C.S., Physician to the General Lunatic Asylum near Nottingham; late of his Majesty's 7th Regiment of Dragoon Guards, and author of "an Essay on Delirium Tremens," &c.

As a malignant species of Cholera prevails epidemically in these kingdoms, and ravages with relentless severity many of our principal towns, it may be well to say a few words concerning its nature and causes. In considering the latter, however, we shall not enter minutely into the much-contested question of contagion; it will, I hope, suffice to say, that we possess abundant proofs of the extensive diffusion of cholera in a manner independent of, and incompatible with, such a mode of its propagation: thus, we see it attack, in the course of a single night, a vast proportion of persons residing in the same district or street; when, as suddenly, the disease will cease, perhaps only to reappear with greater violence within a short space of time, exhibiting evidence of its not having exhausted, during its previous visit, the store of subjects who were predisposed to the disease. This disorder, at least in Europe, is allowed to consist, generally speaking, of three stages: the premonitory, or diarrhœal stage; the choleric, or cold stage; and the febrile stage. It has, likewise, been agreed upon by practitioners, that remedial treatment during the premonitory, or first stage, will succeed, in many instances, in arresting its progress, and in preventing thereby the accession of its other stages. This fact is in itself sufficient to prove that it is not governed by the same laws as those which regulate contagious diseases, properly so called, as we find that nothing can arrest the progress of plague, smallpox, measles, &c.; although their course may be modified very considerably by suitable preparation and treatment; but smallpox, when once induced, will pursue its course to the end. The regularity in the laws of nature, and the dissimilarity in the circumstances mentioned, between cholera and diseases of a decidedly contagious character, dispose me to believe, with Dr. James Johnson, that its ordinary mode of propagation is not by contagion; and, in fact, that it is a non-contagious disease, except under contingent circumstances. Cholera has invariably, at the commencement of its prevalence in the various places in which it has prevailed, confined its ravages to the lowest and most unwholesome situations, such as those who are nearly on a level with the sea, the banks of rivers, &c.; such localities being most likely to abound with decomposable matter. Should more elevated situations become the site of the disease, it will be found that they are either immediately within the sphere of action of emanations arising from the places just spoken of, (in which case, they may become even more unhealthy than the lower grounds from which they arose), or they will be found to contain within themselves sufficient materials to furnish malaria.

It has been frequently observed, that not only concave surfaces, but plains which have no direct fall to carry off water, although in very elevated districts, may become, under certain influences, (which, however, we but imperfectly understand,) the source of miasmal exhalations, and be productive of febrile affections of great malignity. This unrelenting scourge seems, likewise, generally speaking, to select for its victims the poor, the sickly, and the profligate, whose blood has been deteriorated by dissipation, bad living, and constant exposure to vitiated air; the effects of which render individuals liable, on the slightest contingent circumstances, to an attack of the disease; while persons in the higher ranks of life are capable of resisting it, until, by their long residence within the sphere of choleric influence, the nervous system, and, through it the functions generally, become impaired, and thence the system predisposed to the development of cholera: under such circumstances, it will be found to attack the rich as well as the poor. This, however, is no proof of contagion, as the long continuance of epidemic causes which are known to be highly sedative and depressive of nervous power, must gradually lower the nervous energy, and with it the vigour of the various secretory organs; the effect of which is, the necessary deterioration of the quality of the blood, and a consequent predisposition to choleric disease. In this peculiar state, a trifling error in diet may be the exciting cause; or, perhaps, exposure to concentrated emanations from diseased bodies may be sufficient to turn the scale of health, and destroy the balance already more than on the turn. In this manner we see how the disease gradually creeps from the hovels of the poor even to the mansions of the great; and how, in proportion to the duration of the prevalence of cholera, it becomes important for persons in the higher ranks of life to live regularly, and to pay particular attention to the state of their secretions and evacuations; observance of these precautions will, in most instances, prevent predisposition to cholera, and hence the occurrence of the disease itself: for it has been stated, and I believe with reason, that no one in health is susceptible of taking the disease; which is, to say the least, not in favor of the doctrine of contagion. Cholera has been observed to force its way through the strongest sanatory cordons, to travel against the trade winds, and even to gain admittance into our own prisons and workhouses: from which places it ought to have been excluded, were it necessary for its introduction that one of their inmates should have been exposed to the contact of a person labouring under the disease. We shall, therefore, take it for granted, that cholera is induced, at least in the first instance, by a modification of the causes which are productive of fever in general: like common fever, it has its premonitory and cold stages, while its third stage often partakes of the congestive form of typhus itself. These causes, however, appear to be developed very gradually, though steadily, in our climate, and to act slowly on the constitution; depressing the nervous power by degrees, so as not to alarm the ganglionic system, and thus, not interrupt suddenly the general functions of the system; although they may, under such influence, be so far deranged as to be unable to carry on healthy secretion, and thereby permit the accumulation of impure principles in the blood. The deterioration of the blood in quality, which is the effect of this inaction in the liver, kidneys, lungs, &c., is productive of impure and irritating secretions from the mucous linings of the stomach and intestines; hence the diarrhœa, which is an effort of nature to purify the blood, by ridding it of its impurities, and is generally the precursor of cholera, properly so called, constituting what has been termed the first stage of choleric fever. At this period of the disease, the fluids have become unfit to support sthenic action, so that any increase of miasmal influence may so overpower the system generally as to render it unable to propel the blood to the surface of the body; and in this way induce general collapse, attended with accumulation or congestion of blood in the centre of the body, with all the symptoms of true blue or Algid cholera, or the second stage of choleric fever. Should the constitution at this critical juncture rally from this dreadful attack, it will be found unable at first to carry on healthy action, and may, therefore, institute remittent efforts to re-establish health; hence the typhoid fever, which in this country succeeds in so many instances to cholera, constituting the third and last stage of choleric fever. In India, the disease manifests generally but one stage, or that which is termed the Malignant Asiatic Cholera, being but seldom preceded by diarrhœa, or followed by fever. The causes in tropical climates are so intense and sudden as to have the power of at once inducing what we call the second stage of the disease; and as the blood has not, in general, been much deteriorated in quality by long and gradual exposure to the causes of cholera, the system is more frequently enabled at once to emerge from the disease into convalescence. Considering, then, that cholera arises, in the first instance, from local or endemic causes, the most natural and philosophical mode of arresting its progress will be immediately to remove, on the appearance of a case, all the inhabitants from the house in which it occurs to a healthy and distant situation, and to keep them there for a given time, until the causes shall have been removed, either by hygienic measures or the exhausting influence of time. Should a particular street or district become infected generally, it would be desirable to remove all their inhabitants by hutting or encamping them, if no better accommodation could be procured; but their removal from infecting influence will prove the surest mode of saving life. Our next prophylactic measure should be to recommend the strictest attention to the mode of living, by taking wholesome and tonic food, and a rigid observance of cleanliness, ventilation, and warm clothing; by these means we ensure the quality of the ingesta. We should then turn our attention to the nature of the egesta: this includes all the secretions and ex-secretions; the principal of which are those which arise from the liver, intestines, and kidneys. It would be presumptuous in me to attempt to give any directions to attain this object; the means are known to all medical men, who are, I am sure, well convinced of the importance of the subject.

Having thus done all that is within our power to prevent the spreading of the disease, we should endeavour to render it less fatal to those who may labour under it; and for this purpose the treatment of this appalling malady ought to be directed with reference to its causes, and their effects on the system. Much may be done in the premonitory stage, by comparatively simple remedies, and throughout the whole course, we shall find that, if we imitate nature in her efforts to combat it, we shall have followed the safest and most rational mode of practice. This is best done by emulging the secretory organs, with a view to the purification of the blood, by allaying irritation, by counteracting congestion, and by supporting the *vis vitæ,* in every sense of the expression, as well by external applications as by internal means. Should emulging the liver and intestines, on which my chief reliance is placed, in conjunction with the other remedial means alluded to generally, not prove sufficient to correct the deteriorated state of the blood, and prevent the supervention of collapse, we may, as a *dernière ressource*, have recourse to saline injections into the veins, although the operation in itself is attended with considerable risk. The theory by which their action is explained is plausible and philosophical, as their operation tends in a direct way to restore the lost properties of the blood. I should, however, fear the consequence of introducing so large a quantity of fluid as has been injected into the veins, without, at the same time, abstracting a proportionate quantity of the deteriorated blood, either by venesection or the application of leeches, lest artificial plethora might be induced, and with it the suspension of many important functions of the animal economy. I regard the introduction of saline solutions into the veins as a more direct method of restoring the properties which chemistry has proved to be deficient in the blood of cholera patients, than the administration of saline medicines through the medium of the primæ viæ, as we are aware that the secretions from these parts already abound with saline matter; indeed, it has been aptly observed, that such practice is only as if we "carried coals to Newcastle," and incapable of having any beneficial influence on the system; the function of absorption being suspended during the state of collapse. I think it may not be useless, also, to call the attention of practitioners to the possible effects of too sudden and too free an application of heat to the surface of the body during the cold stage of cholera, which might induce a sudden cessation of vitality in the capillaries; on the principle of frost-bitten parts requiring the application of snow rather than warmth to restore action in them. We ought also to be cautious in the administration of stimuli internally, lest, by their action on the mucous linings of the stomach and intestines, which are generally in a highly congested state, we excite an action incompatible with their vitality. I shall venture one more observation in the form of a caution: it is against the unsparing hand with which I have witnessed opium given in this complaint. We ought to remember that cholera is the result of sedative causes, and that, as opium in large doses acts as a sedative, we only increase debility and congestion by their administration; on the contrary, in small and repeated quantities opium will prove a soothing stimulus; and hence a valuable remedy in the treatment of this truly "*opprobrium medicorum.*"

As the object of this appendix is only to endeavour to explain the physiology of cholera on the principles expounded in this essay, I have thought it right to confine myself as much as possible to general observations on the subject, and to avoid entering deeply into the minutiæ of its treatment.

*Nottingham; November* 1832.

